# Detection of SARS-CoV-2 in Neonatal Autopsy Tissues and Placenta 

**DOI:** 10.3201/eid2803.211735

**Published:** 2022-03

**Authors:** Sarah Reagan-Steiner, Julu Bhatnagar, Roosecelis B. Martines, Nicholas S. Milligan, Carly Gisondo, Frank B. Williams, Elizabeth Lee, Lindsey Estetter, Hannah Bullock, Cynthia S. Goldsmith, Pamela Fair, Julie Hand, Gillian Richardson, Kate R. Woodworth, Titilope Oduyebo, Romeo R. Galang, Rebecca Phillips, Elizaveta Belyaeva, Xiao-Ming Yin, Dana Meaney-Delman, Timothy M. Uyeki, Drucilla J. Roberts, Sherif R. Zaki

**Affiliations:** Centers for Disease Control and Prevention, Atlanta, Georgia, USA (S. Reagan-Steiner, J. Bhatnagar, R.B. Martines, E. Lee, L. Estetter, H. Bullock, C.S. Goldsmith, P. Fair, K.R. Woodworth, T. Oduyebo, R.R. Galang, D. Meaney-Delman, T.M. Uyeki, S.R. Zaki);; Tulane University School of Medicine, New Orleans, Louisiana, USA (N.S. Milligan, E. Belyaeva, X.-M. Yin);; Oschner Health, New Orleans (C. Gisondo, F.B. Williams, R. Phillips);; Oak Ridge Institute for Science and Education, Oak Ridge, Tennessee, USA (E. Lee);; Synergy America, Inc., Duluth, Georgia, USA (L. Estetter, H. Bullock);; Louisiana Department of Health, Baton Rouge, Louisiana, USA (J. Hand, G. Richardson);; Massachusetts General Hospital, Boston, Massachusetts, USA (D.J. Roberts)

**Keywords:** COVID-19, 2019 novel coronavirus disease, coronavirus disease, severe acute respiratory syndrome coronavirus 2, SARS-CoV-2, vertical transmission, viruses, respiratory infections, zoonoses, autopsy, infant, newborn, pregnancy

## Abstract

Severe coronavirus disease in neonates is rare. We analyzed clinical, laboratory, and autopsy findings from a neonate in the United States who was delivered at 25 weeks of gestation and died 4 days after birth; the mother had asymptomatic severe acute respiratory syndrome coronavirus 2 (SARS-CoV-2) infection and preeclampsia. We observed severe diffuse alveolar damage and localized SARS-CoV-2 by immunohistochemistry, in situ hybridization, and electron microscopy of the lungs of the neonate. We localized SARS-CoV-2 RNA in neonatal heart and liver vascular endothelium by using in situ hybridization and detected SARS-CoV-2 RNA in neonatal and placental tissues by using reverse transcription PCR. Subgenomic reverse transcription PCR suggested viral replication in lung/airway, heart, and liver. These findings indicate that in utero SARS-CoV-2 transmission contributed to this neonatal death.

Severe acute respiratory syndrome coronavirus 2 (SARS-CoV-2) infection during pregnancy is associated with severe maternal coronavirus disease (COVID-19) and preterm birth and may increase the risk for other complications of pregnancy (*1*–*5*). Although possible vertical SARS-CoV-2 transmission has been reported (*4*,*6*–*8*), the strength of supportive laboratory evidence varies. Risk for SARS-CoV-2 infection in neonates seems to be low, and severe COVID-19 in neonates seems rare (*2*–*4*,*9*). We describe the detection and localization of SARS-CoV-2 in autopsy tissues from a 25-week neonate who died at 4 days of age with clinical history, laboratory, and pathologic findings consistent with severe COVID-19. Asymptomatic SARS-CoV-2 infection was diagnosed for the infant’s mother after universal screening and preeclampsia.

## Clinical History of the Mother

The mother was a 34-year-old woman in the United States with a history of 3 prior pregnancies that resulted in live births. She was severely obese (prepregnancy body mass index 47.5 kg/m^2^) and had chronic hypertension and a history of preeclampsia in 2 prior pregnancies. She was hospitalized at 25 weeks of gestation for preeclampsia management. Other than systolic blood pressure >160 mm Hg and proteinuria, she was otherwise asymptomatic. She received routine prenatal care starting in the first trimester, and her blood pressure was well controlled until 24 weeks of gestation. At the time of hospitalization, she received magnesium sulfate, intravenous antihypertensive medications, and betamethasone for fetal lung maturation. On hospital day 2, a nasopharyngeal swab sample collected for SARS-CoV-2 screening by reverse transcription PCR (RT-PCR) tested positive. The pregnancy had occurred before COVID-19 vaccines were available in the United States. The patient reported no known SARS-CoV-2 exposures, previous SARS-CoV-2 testing, or COVID-19 symptoms. She had completed her antenatal regimen of corticosteroids, and fetal assessment remained reassuring. However, on day 5, an urgent cesarean delivery was performed because of preeclampsia with severe features. Delivery and maternal postpartum course were uncomplicated. The patient was discharged on day 9 and did not subsequently experience fever or respiratory symptoms. No subsequent SARS-CoV-2 testing was performed.

## Clinical History of the Neonate

The male infant, delivered at 25 weeks and 6 days of gestation, was immediately taken to a radiant warmer without any maternal contact. Apgar scores were 1 at 1 minute, 4 at 5 minutes, and 7 at 10 minutes. He was intubated within 5 minutes of birth. His birth weight was 670 g (16th percentile), length 32.5 cm (33rd percentile), and head circumference 21.5 cm (6th percentile).

In the neonatal intensive care unit, the neonate was immediately placed under airborne, contact, and droplet precautions in a single-patient room. A chest radiograph showed diffuse bilateral granular opacities without focal consolidation. He received oxygen, an intratracheal dose of surfactant, parenteral nutrition, caffeine, and prophylactic fluconazole. A complete blood count revealed a leukocyte count of 3,150 cells/μL (reference range of 9,000–30,000 cells/μL), a hematocrit of 40.7% (reference range 42%–63%), and a platelet count of 114,000/μL (reference range 150,000–350,000 cells/μL).

At 1 day of age, the neonate was extubated and positive-pressure ventilation was administered; however, by 2 days of age, he was reintubated because of worsening respiratory status and consolidative changes on chest radiograph. A second dose of surfactant was given, and a packed red blood cell transfusion was given because of a hematocrit of 29%. A chest radiograph taken ≈12 hours later showed progression of diffuse bilateral lung opacification and air bronchograms in the lung bases. Oxygenation index was 16.2, demonstrating a severe oxygen deficit consistent with severe neonatal acute respiratory distress syndrome (oxygen index >16) (*10*).

 At 3 days of age, cardiopulmonary resuscitation was initiated for bradycardia; fluids, vasopressors, and hydrocortisone were administered for hypotensive shock. Vancomycin and amikacin were empirically initiated. A complete blood count revealed 850 leukocytes/μL, 85 neutrophils/μL (reference range 1,300–15,000 neutrophils/μL), and platelets 3,000/μL, for which a platelet transfusion was given. Results of SARS-CoV-2 RT-PCRs on nasopharyngeal swab samples collected at 24 and 72 hours after delivery were positive. Bacterial blood and endotracheal aspirate cultures collected at 3 days of age were negative.

When the neonate was 4 days of age, ventilator and vasopressor requirements increased. A chest radiograph showed continued widespread bilateral airspace consolidation, and oxygenation index was 46.7. Phenobarbital was given for possible seizure activity, and cefepime was added. Despite increasing ventilator support, respiratory acidosis worsened, and an acute bradycardic event occurred. Death was pronounced at 4 days of age, and parental consent for autopsy was obtained.

## Postmortem and Placenta Examinations 

A complete autopsy and placental examination were performed per standard protocol at the clinical institutions. Formalin-fixed, paraffin-embedded (FFPE) neonatal lung, airway, heart, liver, spleen, and kidney tissues and placental tissues were submitted by the clinical institutions to the Infectious Diseases Pathology Branch, Division of High-Consequence Pathogens and Pathology, National Center for Emerging and Zoonotic Infectious Diseases, Centers for Disease Control and Prevention (CDC), for diagnostic consultation along with medical and autopsy records. This activity was reviewed by CDC and conducted consistent with applicable federal law and CDC policy (45 C.F.R. part 46; 21 C.F.R. part 56; 42 U.S.C. §241(d); 5 U.S.C. §552a; 44 U.S.C. §3501 et seq.).

At CDC, we performed routine hematoxylin-eosin staining for histopathologic evaluation and Gram and Grocott methenamine silver staining to evaluate for bacterial and fungal pathogens. We performed immunohistochemistry (IHC) for SARS-CoV-2 viral antigens (nucleocapsid and spike proteins) as previously described (*11*) as well as angiotensin-converting enzyme 2 (ACE2), transmembrane serine protease 2 (TMPRSS2), and CD163 IHC assays. We performed SARS-CoV-2 conventional RT-PCR and sequencing on RNA extracted from FFPE tissues, as previously described (*12*). We also performed subgenomic RNA RT-PCR and in situ hybridization (ISH) on samples positive by conventional RT-PCR (*12*) and selected only areas with abundant IHC or ISH staining for electron microscopy (*11*).

### Histopathology and Immunohistochemistry Assays

For SARS-CoV-2 IHC, we used rabbit monoclonal SARS-CoV-2 nucleocapsid HL448 antibody (GTX635686) and mouse monoclonal SARS-CoV-2 spike S1 antibody (GTX635654; both from GeneTex, https://www.genetex.com). We also used ACE2 goat polyclonal antibody (R&D Systems, https://www.rndsystems.com), transmembrane serine protease 2 (TMPRSS2) rabbit polyclonal PA5-76776 (Thermo Fisher Scientific, https://www.thermofisher.com), and CD163 mouse monoclonal antibody (clone 10D6; Leica Biosystems, https://www.leicabiosystems.com) for IHC. Double-stained IHC assays were performed according to manufacturer guidelines by using the mouse monoclonal SARS-CoV-2 spike S1 antibody and CD163 mouse monoclonal antibody with the TripleStain IHC Kit: M&M&R on human tissue (DAB, AP/Red & HRP/Green, ab183286; abcam, https://www.abcam.com).

### RT-PCR, Sequencing, and ISH Assays

We extracted RNA from FFPE autopsy and placental tissues by using the phenol-chloroform extraction protocol, as previously described (*13*) and evaluated all samples by using 2 conventional RT-PCR assays targeting the spike (S) and nucleocapsid (N) genes for SARS-CoV-2. The assays were performed by using the OneStep RT-PCR Kit (QIAGEN, https://www.qiagen.com) and 5 μL of RNA sample. The N-gene (150-bp) and S-gene (162-bp) amplicons positive by PCR were directly sequenced by Sanger sequencing on a GenomeLab GeXP sequencer (AB SCIEX, https://sciex.com). We searched for homologies to known sequences by using the BLAST nucleotide database (https://blast.ncbi.nlm.nih.gov/Blast.cgi). To demonstrate evidence of probable viral replication, we performed subgenomic RNA RT-PCR (*14*). To directly localize SARS-CoV-2 RNA, we performed ISH assays targeting the N and S genes on FFPE tissues that were positive for SARS-CoV-2 by conventional RT-PCR.

## Postmortem and Placenta Findings 

### Neonate 

**Figure 1:**
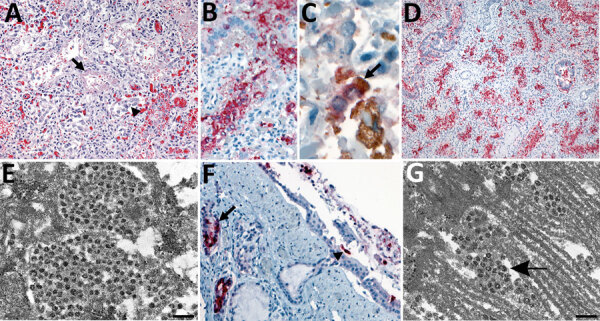
Pulmonary histopathologic, immunohistochemical (IHC), in situ hybridization, and ultrastructural findings in tissues from a neonate in the United States with severe acute respiratory syndrome coronavirus 2 (SARS-CoV-2). A) Lower magnification of the lung showing diffuse alveolar damage, characterized by type II pneumocyte hyperplasia (arrowhead), hyaline membrane (arrow), and interstitial mononuclear infiltrate. Original magnification ×20. B) Extensive intra-alveolar immunostaining by spike protein SARS-CoV-2 IHC assay. Original magnification ×40. C) Double-stain IHC assay showing rare macrophages with SARS-CoV-2/CD-163–positive immunostaining. Red, SARS-CoV-2; brown, CD-163 antibody (arrow). Original magnification ×63. D) Extensive staining of SARS-CoV-2 genomic RNA in pneumocytes by nucleocapsid gene in situ hybridization assay. Original magnification ×10. E) Electron microscopy (EM) image of a pneumocyte containing accumulations of intracellular viral particles. Scale bar indicates 200 nm; viral particles were on average 65 nm in diameter, smaller than commonly observed because of shrinkage during processing. F) Immunostaining of tracheal epithelial cells (arrowhead) and submucosal glands (arrow) by SARS-CoV-2 nucleocapsid IHC assay. Original magnification ×20. G) EM image of a ciliated epithelial cell with extracellular viral particles (arrow) associated with the cilia. Scale bar indicates 200 nm. EM images were collected from 4-μm sections of formalin-fixed, paraffin-embedded tissues affixed to glass slides that were embedded for EM.

**Figure 2:**
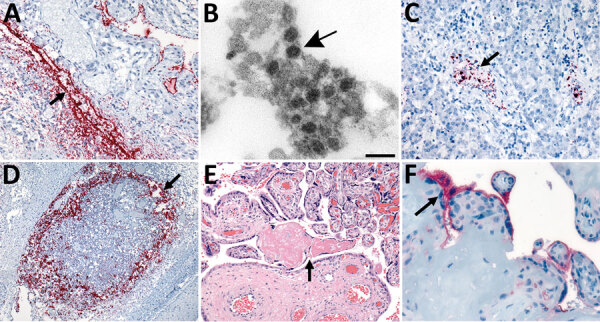
In situ hybridization (ISH) slides demonstrating localization of severe acute respiratory syndrome coronavirus 2 (SARS-CoV-2) genomic RNA in heart, liver, and lymph node tissues and electron microscopic evidence of viral particles in heart tissue from neonate in the United States that died with SARS-CoV-2 infection and placental histopathology and angiotensin-converting enzyme-2 immunohistochemical stain slides. A) SARS-CoV-2 RNA staining by nucleocapsid gene ISH assay in the endothelial cells in myocardium vessel walls (arrow). Original magnification ×20. B) Extracellular virus particles in the connective tissue of the heart (arrow). Scale bar indicates 100 nm. C) Intravascular staining by nucleocapsid gene ISH assay in the liver parenchyma (arrow). Original magnification ×20. D) Extensive nucleocapsid gene ISH staining within macrophages of subcapsular sinus of lymphoid follicle in the submucosa of upper airway (arrow). Original magnification ×10. E) Second trimester placenta with fibrinoid necrosis (arrow). Original magnification ×20. F) Angiotensin-converting enzyme 2 immunostaining in the membrane polarized on the maternal lake side in the syncytiotrophoblast (arrow). Original magnification ×63.

Microscopic examination of lungs from the neonate showed peripheral vascularization consistent with 24–26 weeks of gestation. We observed severe diffuse alveolar damage with hyaline membranes, type II pneumocyte hyperplasia, and mild interstitial mononuclear infiltrate (Figure 1, panel A). We found no histopathologic evidence of bronchopneumonia; Gram staining revealed no bacterial pathogens; and Grocott methenamine silver staining revealed no fungal pathogens. We identified SARS-CoV-2 viral antigens in alveolar macrophages, type II pneumocytes (Figure 1, panel B), and hyaline membranes. Rare macrophages demonstrated SARS-CoV-2/CD163 double-staining (Figure 1, panel C). ISH demonstrated viral RNA in alveolar macrophages and pneumocytes (Figure 1, panel D). We observed viral antigens (Figure 1, panel F) and RNA in airway, bronchiolar, and submucosal gland epithelium and in macrophages in prominent airway submucosa lymphoid follicles (Figure 2, panel D).

Although we observed no significant histopathologic findings in extrapulmonary tissues, ISH detected SARS-CoV-2 RNA in vascular endothelial cells in the myocardium (Figure 2, panel A), where viral antigens were also observed, and in the liver (Figure 2, panel C). IHC assays of the liver and IHC and ISH of the spleen and kidney produced negative results.

SARS-CoV-2 RNA was detected in lung, airway, heart, liver, spleen, and kidney tissue by conventional RT-PCR; sequence analysis of PCR amplicons showed 99%–100% nt identity with SARS-CoV-2. Subgenomic RNA, suggesting SARS-CoV-2 replication (*12*), was detected by subgenomic RT-PCR in lung, airway, heart, and liver tissue but not in spleen or kidney tissue.

Electron microscopy revealed coronavirus-like particles in areas corresponding to SARS-CoV-2 IHC and ISH staining in respiratory and myocardial tissues. We found vacuolar accumulations of coronavirus particles within pneumocytes (Figure 1, panel E) and extracellular viral particles in association with cilia of respiratory epithelial cells (Figure 1, panel G) and near collagen in the heart (Figure 2, panel B).

### Placenta 

The trimmed placenta was 72 g, which was small for gestational age (10th percentile weight for a singleton placenta at 25 weeks gestation would be ≈159 g) (*15*). We found microscopic evidence of maternal vascular malperfusion, including placental hypoplasia, accelerated villous maturation, and focally increased perivillous fibrin (Figure 2, panel E). We also noted increased villous fibrinoid necrosis, a feature of villous trophoblastic injury. We observed low-grade fetal vascular malperfusion with multifocal avascular villi but no villitis or histiocytic intervillositis. ACE2 was multifocally expressed in the syncytiotrophoblast and cytotrophoblast in the maternal lake side and in the decidua basalis (Figure 2, panel F). Weak TMPRSS2 staining was observed in the syncytiotrophoblast membrane. Placental parenchyma was positive by SARS-CoV-2 conventional RT-PCR, confirmed by sequencing but negative by subgenomic RT-PCR, IHC, and ISH. Trivascular umbilical cord and fetal membranes were unremarkable, and all SARS-CoV-2 assay results for cord and membrane samples were negative.

## Discussion

We provide direct evidence of SARS-CoV-2 infection and probable viral replication in multiple autopsy tissues from a premature infant who died with severe COVID-19. Our findings are most consistent with virus acquisition via in utero transmission. We found extensive staining of SARS-CoV-2 antigens and RNA and evidence of plausible virus replication in the lungs, which demonstrated significant pathology. Heart and liver demonstrated vascular endothelial RNA staining and subgenomic RT-PCR positivity, consistent with hematogenous dissemination to the primary targets of fetal circulation and probable virus replication in these organs. Although other mechanisms of vertical transmission cannot be definitively excluded, placental positivity by conventional RT-PCR suggests that SARS-CoV-2 RNA was in the maternal circulation, and transplacental transmission could have occurred only if maternal viremia was present before delivery. Furthermore, although the incubation period after in utero SARS-CoV-2 exposure is unknown, development of advanced pulmonary pathology, including diffuse alveolar damage with extensive staining of viral antigens and RNA, and extrapulmonary dissemination of SARS-CoV-2 would be unlikely if transmission occurred intrapartum or postnatally.

Viral infections during pregnancy and after delivery can lead to infant illness and death (*16*,*17*). Vertical transmission of viruses can occur in 3 ways: 1) in utero (via maternal viremia and either placental cell infection or placental barrier disruption), 2) intrapartum (from maternal body fluids during birth), or 3) postnatally (e.g., from breastfeeding, caregiver exposures) (*16*,*17*). Thus far, reports consistent with in utero SARS-CoV-2 transmission have been rare and include mother–infant pairs with evidence of SARS-CoV-2 in maternal specimens (e.g., placenta) and neonatal specimens (e.g., respiratory swab samples collected <24 hours postnatally) (*18*,*19*). Although a review of 176 neonatal SARS-CoV-2 infections reported in the literature estimated that ≈30% could have resulted from vertical transmission, those data were not based on systematic testing or surveillance activities (*8*).

Vertical transmission of SARS-CoV-2 and severe neonatal COVID-19 seem infrequent; however, risk for medically indicated preterm delivery and stillbirth among women with SARS-CoV-2 infection during pregnancy seems to be elevated (*2*,*3*,*7*,*8*). Although severe respiratory disease in SARS-CoV-2–positive late preterm or term neonates has been reported (*4*–*7*), we detected SARS-CoV-2 and evidence of probable virus replication in autopsy tissues from an extremely preterm neonate. In addition, maternal SARS-CoV-2 infection occurred during the first or second trimester. Most reports of SARS-CoV-2 infection during pregnancy describe infection in the third trimester (*1*–*5*). Additional data on outcomes among women with first or second trimester SARS-CoV-2 infection, including data specifically for preterm infants (*2*–*4*,*9*), are needed.

Given the extreme prematurity of this infant, the relative contributions of neonatal respiratory distress syndrome versus SARS-CoV-2 infection to the observed lung pathology and patient outcome are difficult to disentangle. However, abundant staining of SARS-CoV-2 antigens and RNA in the lungs and evidence of probable virus replication in the context of pathology typical of COVID-19 in adults (*11*,*12*) indicate that SARS-CoV-2 infection played a central role in this case. Furthermore, the neonate’s condition did not improve after repeated surfactant administration.

Placental cells express SARS-CoV-2 ACE2 and TMPRSS2 receptors, as in this case, and the genes necessary for viral replication (*20*–*23*). However, receptor density and colocalization vary throughout pregnancy, potentially leading to differential risk for placental infection by trimester (*23*,*24*). In addition, SARS-CoV-2 RNA is rarely detected in the placenta, and electron microscopy has misidentified common subcellular structures as coronavirus particles (*25*–*28*). In this patient, we found neither evidence of placental SARS-CoV-2 infection by ISH or IHC (*29*–*34*) nor evidence of chronic histiocytic intervillositis, which has been identified as a relatively consistent pathologic feature associated with placental SARS-CoV-2 infection (*32*–*34*). Consequently, conventional RT-PCR positivity may represent maternal viremia.

Other case series have described placental SARS-CoV-2 detection in the syncytiotrophoblast, cytotrophoblast, Hofbauer cells, and villous endothelial cells, including in some cases with evidence of in utero SARS-CoV-2 transmission (*18*,*32*–*34*). Although the timing of maternal infection cannot be established in this case, if the infection was acute and viremia was present, it is possible that substantial placenta pathology had not yet developed or may have been missed during placenta sampling. Hematogenous in utero transmission of some viral infections also occurs in the absence of placental infection (*35*). Factors such as hypoperfusion and trophoblast ischemic damage or transient maternal–fetal hemorrhage could have exposed the villus stroma to maternal blood and led to SARS-CoV-2 transfer to fetal circulation without placental cellular infection. The relationship between SARS-CoV-2 infection, preeclampsia, and maternal and infant outcomes is complex. Although the rate of preeclampsia might be elevated among pregnant women with COVID-19 (*2*,*5*), the effect of SARS-CoV-2 infection on its development or severity, particularly in patients with multiple preeclampsia risk factors, is unknown.

The World Health Organization and others have proposed definitions for in utero SARS-CoV-2 transmission (*16*,*17*,*31*). This case would meet World Health Organization criteria for possible in utero SARS-CoV-2 transmission; however, it would not meet the definition of confirmed transmission because virus persistence criteria were not met (i.e., RT-PCR SARS-CoV-2 positivity for a sterile sample at 24–48 hours of life) (*16*). Adding criteria for neonatal autopsy tissue–based molecular evidence of SARS-CoV-2 could be useful, similar to criteria for establishing in utero transmission for a fetal demise.

This case demonstrates that in utero SARS-CoV-2 transmission is possible and can lead to serious outcomes for infants. Further work is needed to provide more information about risk factors for mother-to-child transmission, adverse pregnancy outcomes, and infant outcomes in women with SARS-CoV-2 infection during pregnancy and to inform patient management and testing strategies, prevention, and individual COVID-19 vaccination decision making. COVID-19 vaccination before or during pregnancy is strongly recommended by CDC, the American College of Obstetrics and Gynecology, and the Society for Maternal-Fetal Medicine (*36*–*38*). However, vaccination uptake among pregnant women is currently low in the United States; as of September 27, 2021, only 31% of pregnant women were fully vaccinated before or during pregnancy (*39*). Continued public health surveillance for pregnancy and infant outcomes by trimester of SARS-CoV-2 infection is warranted, including evaluation of placental, fetal, or infant specimens from COVID-19–affected pregnancies when possible and clinically indicated. SARS-CoV-2 testing during pregnancy should be guided by routine assessment for COVID-19–associated signs/symptoms and exposures, presence of complications potentially associated with SARS-CoV-2 infection (e.g., preeclampsia) (*2*,*5*), and level of community transmission. Neonates born to women with suspected or confirmed COVID-19, regardless of neonatal signs/symptoms, should also be tested for SARS-CoV-2 (*40*).
